# TGFB2-mediated regeneration of pelvic ligament equivalents using a stem cell-fibroblast-decellularized membrane composite

**DOI:** 10.3389/fbioe.2026.1763991

**Published:** 2026-07-23

**Authors:** Yingying Dong, Yu Zhang, Yinan Duan, Zhijun Xia

**Affiliations:** 1 Department of Obstetrics and Gynecology, Shengjing Hospital of China Medical University, Shenyang, Liaoning, China; 2 Department of Gynecology, Liaoning Maternal and Child Health Hospital, Shenyang, Liaoning, China; 3 Department of Research and Development, Shenyang Engineering Technology R&D Center of Cell Therapy Co., Ltd, Shenyang, Liaoning, China; 4 Department of Gynecology, Obstetrics and Gynecology Hospital of Fudan University, Shanghai, China

**Keywords:** adipose-derived stem cells, decellularized human amniotic membrane, extracellular matrix, pelvic organ prolapse, tissue engineering

## Abstract

Pelvic organ prolapse (POP) is a prevalent condition characterized by weakened pelvic floor tissues, significantly impacting women’s quality of life. Current treatments, including synthetic meshes and native tissue repair, face challenges of high recurrence rates and complications. This study developed a novel tissue-engineered strategy utilizing a decellularized human amniotic membrane (HAAM) scaffold seeded with autologous adipose-derived mesenchymal stem cells (ADSCs) and vaginal wall fibroblasts (HVFs) to construct a bioactive pelvic ligament equivalent. We first validated the successful preparation of HAAM with preserved extracellular matrix integrity and characterized the phenotypic markers of ADSCs and HVFs. In a rat abdominal wall defect model, the HAAM+ADSCs+HVFs composite demonstrated superior tissue regeneration and integration, reduced fibrosis, and effective modulation of the host immune microenvironment —evidenced by enhanced repair and decreased infiltration of pro-inflammatory cells —compared to all control groups (including HAAM alone and single-cell groups). Transcriptomic analysis revealed that the composite treatment promoted extracellular matrix organization and collagen synthesis while suppressing matrix degradation (MMP2/MMP9) and inflammatory pathways. Most importantly, mechanistic studies identified TGFB2 as the key paracrine mediator through which ADSCs exert their effects: TGFB2 inhibition abolished ADSCs-induced improvements in HVFs proliferation, migration, and anti-apoptosis, while its overexpression replicated these benefits. Western blot confirmed that TGFB2 coordinately upregulates COL1A1/COL3A1 and downregulates MMP2/MMP9 to restore ECM homeostasis. These comprehensive findings demonstrate that the HAAM-based ADSCs-HVFs composite promotes functional tissue regeneration via TGFB2-mediated mechanisms, offering a safe, effective, and regenerative therapeutic strategy with strong translational potential for POP.

## 1 Introduction

Pelvic organ prolapse (POP) is a multifactorial condition characterized by the weakening of pelvic floor supportive tissues, leading to the descent of pelvic organs. As a prevalent form of pelvic floor dysfunction, POP is associated with risk factors such as childbirth, aging, and obesity. With a global aging population, POP has evolved into a major public health concern, severely impacting women’s quality of life and imposing a substantial socioeconomic burden. It is predicted that by 2050, the prevalence of POP in the United States will increase to 46%, affecting over 50 million individuals (Wu et al., 2009). In China, epidemiological data indicate a symptomatic POP prevalence of 9.6%, which escalates markedly with age, reaching 28.21% in women over 70 (Pang et al., 2021). When conservative management fails, surgical intervention becomes the mainstay, underscoring a vast and growing clinical need for effective POP reconstruction.

Currently, POP reconstructive surgeries are mainly divided into native tissue repair and mesh implantation. However, native tissue repair is hampered by high recurrence rates (30%–40% at 5 years), prompting the widespread adoption of polypropylene mesh. Although initially promising for its mechanical strength and efficacy, the non-degradable polypropylene mesh is plagued by severe complications—including erosion, chronic pain, and dyspareunia—due to poor tissue integration and biocompatibility. Consequently, the U.S. FDA banned its transvaginal use for POP in 2019 (FDA 510k Premarket Notification K020643, 2002; FDA. Urogynecologic Surgical Mesh, 2019). This regulatory action has left a critical void in the clinical arsenal, highlighting an urgent, unmet need for safer and more effective regenerative solutions. In this context, tissue engineering strategies aimed at constructing biomechanically competent and biologically active pelvic ligament equivalents have emerged as a pivotal research Frontier (Raya-Rivera et al., 2014).

The success of tissue engineering hinges on two core elements: functional seed cells and an optimal scaffold. Fibroblasts, as the principal cellular component of pelvic ligaments, are responsible for synthesizing and remodeling the extracellular matrix (ECM)—primarily collagen type I (Col-I) and type III (Col-III)—which directly governs tissue strength and elasticity (Moalli et al., 2005). Col-I confers tensile strength, while Col-III imparts flexibility (Chen et al., 2016). Notably, POP pathogenesis is closely linked to ECM disorders, with patients exhibiting significantly reduced total collagen content and altered Col-I/III ratios, compromising ligament integrity (Jackson et al., 1996; Takacs et al., 2008; Liapis et al., 2001). This underscores collagen production as a key indicator for evaluating repair, a process intrinsically dependent on viable and active fibroblasts.

However, vaginal wall fibroblasts (HVFs) derived from POP patients often exhibit a senescent phenotype with diminished proliferative capacity and ECM secretory function (Poncet et al., 2005; Boreham et al., 2002). This intrinsic cellular deficit poses a significant challenge for autologous tissue engineering. Mesenchymal stem cells (MSCs), particularly adipose-derived stem cells (ADSCs), offer a compelling solution. ADSCs are abundant, easily accessible, and possess potent paracrine capabilities. They can secrete a milieu of bioactive factors that promote fibroblast proliferation, collagen synthesis, and modulate the immune microenvironment by polarizing macrophages toward a regenerative M2 phenotype (Dominici et al., 2006; Caplan and Correa, 2011; Mantovani et al., 2013). Therefore, in the context of pelvic floor reconstruction using biological grafts, we propose a synergistic cellular strategy: utilizing autologous ADSCs not merely as alternative seed cells, but as “cellular bio-activators.” Their role is to rejuvenate the dysfunctional, patient-derived HVFs—the authentic ECM-producing workhorses of the pelvic ligament—through paracrine signaling. This co-culture approach aims to transform compromised HVFs into functionally competent seed cells, thereby directly addressing the cellular pathogenesis of POP. This rationale is supported by prior studies on fibroblast-MSC interactions (Ren et al., 2008; Zuk et al., 2001; Kim et al., 2009; Cao et al., 2016).

The choice of scaffold is equally critical. An ideal scaffold must provide temporary mechanical support, exhibit excellent biocompatibility, and actively guide tissue regeneration. The decellularized human amniotic membrane (HAAM) stands out as a promising biological scaffold candidate for pelvic ligament engineering (Mamede et al., 2012). Decellularization effectively removes immunogenic cellular components while preserving the native ECM architecture, including inherent Col-I, Col-III, and growth factors like bFGF (Moalli et al., 2005; Niknejad et al., 2008). Beyond its established biocompatibility and pro-regenerative bioactivity, HAAM holds particular promise as a biological suspensory ligament substitute due to its inherent biomechanical profile. Studies have shown that decellularized amniotic membrane retains considerable tensile strength and elasticity, key mechanical properties required for load-bearing pelvic support. This unique composition endows HAAM with several advantages for constructing a pelvic ligament equivalent: (1) it provides an initial biomechanical scaffold to withstand physiological loads; (2) its bioactive ECM components can directly stimulate seeded cells to proliferate and synthesize functional matrix; and (3) its degradability allows for gradual replacement by neo-tissue, facilitating true regeneration rather than permanent implantation.

In summary, there is a pressing clinical demand for a regenerative biological graft alternative to synthetic meshes in POP repair. This study innovatively proposes a tissue-engineered biological construct combining autologous ADSCs and HVFs seeded onto a HAAM scaffold. We hypothesize that this bio-hybrid system will leverage the paracrine and immunomodulatory effects of ADSCs to revitalize patient-derived HVFs, while the HAAM scaffold provides the necessary structural and biological cues to direct the formation of a functional ligament equivalent. The following work validates this strategy through *in vitro* and *in vivo* models, aiming to lay a robust foundation for a new, safe, and effective therapeutic paradigm for POP.

## 2 Materials and methods

### 2.1 ADSCs extraction and phenotypic identification

ADSCs were extracted from subcutaneous adipose tissue near the puncture site of four symptomatic POP patients (stage III-IV). The study protocol was approved by the Ethics Committee of Liaoning Maternal and Child Health Hospital (Approval No [2022PS154K, 20240830001]). All patients provided written informed consent. The adipose tissue was processed within 2 °C–8 °C transport. After washing with PBS containing 1% penicillin-streptomycin (Gibco, 15140122), it was digested with an equal volume of 0.1% collagenase I (Sigma-Aldrich, C0130) at 37 °C for 60 min. The stromal vascular fraction (SVF) was collected by centrifugation (300g, 10 min), resuspended, and filtered through a 70-μm cell strainer. Cells were cultured in complete growth medium: Dulbecco’s Modified Eagle Medium/Nutrient Mixture F-12 (DMEM/F12, Gibco, 11330032) supplemented with 10% fetal bovine serum (FBS, Gibco, 10099141) and 1% penicillin-streptomycin (Gibco, 15140122) at 37 °C in a humidified atmosphere of 5% CO_2_. Cells at passages 3–5 (P3-P5) were used.

For flow cytometry, P3 cells (1 × 10^6^) were stained with PE-conjugated antibodies against human CD73 (BD Biosciences, 550257), CD105 (BD Biosciences, 560819), CD34 (BD Biosciences, 555822), CD45 (BD Biosciences, 555483), and FITC-conjugated CD90 (BD Biosciences, 555595) according to manufacturer’s instructions. Data were acquired on a SONY SA3800 flow cytometer and analyzed using FlowJo software (v10.8.1).

### 2.2 HVFs extraction and identification

Vaginal wall tissues were obtained from four symptomatic POP patients and four non-POP patients undergoing hysterectomy for benign conditions. This procedure was approved by the same ethics committee (Approval No [2022 S154K, 20240830001]), with informed consent obtained from all participants. Tissues were processed under sterile conditions. The lamina propria was minced into ∼1 mm^3^ explants and adhered to culture dishes for 1 h. Explants were cultured in the same complete growth medium as described for ADSCs (DMEM/F12 with 10% FBS and 1% penicillin-streptomycin) at 37 °C, 5% CO_2_. HVFs at passages 4–6 (P4-P6) were used.

For immunocytochemistry, cells on coverslips were fixed with 4% paraformaldehyde (Servicebio, G1101) and permeabilized with 0.5% Triton X-100. After blocking with 5% BSA, cells were incubated overnight at 4 °C with primary antibodies: mouse anti-Vimentin (1:200, Abcam, ab8978), mouse anti-Cytokeratin 14 (1:200, Abcam, ab7800), and rabbit anti-α-SMA (1:200, Abcam, ab5694). Subsequent steps were performed using a SP (Streptavidin-Peroxidase) IHC Kit (ZSGB-BIO, SP-9001) following the manufacturer’s protocol, with DAB (ZSGB-BIO, ZLI-9018) as the chromogen. PBS replaced primary antibodies for negative controls.

### 2.3 CCK8 cell proliferation assay

HVFs (5 × 10^4^ cells/well) were seeded in 96-well plates. For co-culture, ADSCs were seeded in Transwell inserts (0.4 μm pore size, Corning, 3470) at specified ratios (ADSCs:HVFs = 0.5:1, 1:1, 2:1). The TGF-β inhibitor SB431542 (MedChemExpress, HY-10431) was used at 10 μM. Cells were cultured in DMEM/F12 with 10% FBS. At 24, 48, and 72h, 10 μL of CCK-8 reagent (Dojindo, CK04) was added to each well. After 2 h incubation at 37 °C, absorbance at 450 nm was measured using a microplate reader (BioTek, Epoch). The blank control was medium only. Each condition had three independent replicates (n = 3).

### 2.4 qRT-PCR experimental

Total RNA was extracted using TRIzol Reagent (Invitrogen, 15596026). cDNA was synthesized using the PrimeScript RT reagent Kit (Takara, RR037A). qPCR was performed with TB Green Premix Ex Taq II (Takara, RR820A) on a QuantStudio 6 Flex system (Applied Biosystems). The reaction conditions were: 95 °C for 30s, followed by 40 cycles of 95 °C for 5s and 60 °C for 30s. Primer sequences are listed in Table 1. GAPDH served as the internal control. Relative gene expression was calculated using the 2^(-ΔΔCt) method. Each sample was analyzed in triplicate.

**TABLE 1:** Primer sequences.

Name	Forward primer (5'->3′)	Reverse primer (5'->3′)
Col-I	AGG​GCC​AAG​ACG​AAG​ACA​TC	AGA​TCA​CGT​CAT​CGC​ACA​ACA
Col-III	TGG​CTA​CTT​CTC​GCT​CTG​CTT	CGG​ATC​CTG​AGT​CAC​AGA​CAC​A
GAPDH	CAG​GAG​GCA​TTG​CTG​ATG​AT	GAAGGCTGGGGCTCATTT

### 2.5 Western blot

Total protein was extracted using RIPA Lysis Buffer (Beyotime, P0013B) with protease inhibitors. Protein concentration was determined by BCA assay (Beyotime, P0012). Samples (20 μg) were separated on 8% SDS-PAGE gels and transferred to PVDF membranes (Millipore, IPVH00010). Membranes were blocked with 5% skim milk and incubated overnight at 4 °C with the following primary antibodies (all from Abcam): rabbit anti-TGFB2 (ab227687, 1:1000), rabbit anti-COL1A1 (ab34710, 1:1000), rabbit anti-COL3A1 (ab184993, 1:1000), rabbit anti-MMP2 (ab92536, 1:1000), rabbit anti-MMP9 (ab76003, 1:1000), and mouse anti-β-actin (ab8226, 1:5000). After incubation with HRP-conjugated goat anti-rabbit/mouse secondary antibodies (1:5000, ZSGB-BIO, ZB-2301/ZB-2305), bands were visualized using ECL detection reagents (Millipore, WBKLS0500) and quantified with ImageJ software (v1.53k). Three independent biological replicates were performed.

### 2.6 HAAM preparation and cell adhesion detection

Full-term placentas were obtained from healthy donors following cesarean section with informed consent and ethical approval (Approval No [2022PS154K, 20240830001]). The amniotic membrane was mechanically separated, washed thoroughly with saline, and decellularized by immersion in 1% Triton X-100 (Sigma-Aldrich, X100) with agitation at 37 °C for 6 h. After three 15-min PBS washes, it was treated with 1% trypsin (Gibco, 25200056) overnight at 4 °C. The resulting decellularized human amniotic membrane (HAAM) was cut into 2 × 2 cm^2^ pieces and sterilized under UV light for subsequent use.

For cell-scaffold construct preparation, HVFs, ADSCs, or a 1:1 co-culture (at a density of 5 × 10^5^ cells per scaffold piece) were seeded onto the epithelial side of HAAM placed in 6-well plates. The constructs were cultured in complete growth medium (as described in 2.1) at 37 °C in a humidified incubator with 5% CO_2_ for 3–7 days prior to any further experimentation or *in vivo* implantation. During this period, the medium was changed every 48 h.

Cell adhesion and retention on the HAAM scaffold were rigorously verified. First, cell morphology and distribution were assessed qualitatively by H&E staining after 1, 3, and 7 days of culture. Second, prior to *in vivo* implantation, constructs were gently rinsed with sterile PBS to remove non-adherent cells, ensuring that only well-integrated cells were transplanted. This protocol was designed to maximize cell retention on the scaffold, providing a reliable basis for the subsequent *in vivo* therapeutic efficacy studies.

### 2.7 Experimental method for rat abdominal wall defect-cell scaffold composite repair

We explicitly chose immunocompetent SD rats (rather than immunodeficient models) for this proof-of-concept study for two key reasons: (1) To rigorously evaluate the acute host inflammatory and immunomodulatory response to our biohybrid construct, which is a critical safety parameter for any future clinical translation; and (2) To assess whether the proposed ADSCs-mediated immunomodulation could effectively mitigate xenogeneic rejection in a challenging, immunologically active environment (Approval No [2024PS1066K]).

The experiment utilized 40 healthy 6-week-old female Sprague Dawley (SD) rats, weighing 200 ± 20 g, which were randomly divided into six groups (n = 8 per group). After 1 week of acclimatization, the modeling procedure was initiated. The specific groups were as follows: A) Blank Control group (sham operation, normally fed); B) Model group (abdominal wall defect created, no repair); C) HAAM group (defect repaired with acellular HAAM scaffold alone); D) HAAM + ADSCs group (defect repaired with HAAM scaffold pre-seeded with ADSCs); E) HAAM + HVFs group (defect repaired with HAAM scaffold pre-seeded with HVFs); and F) HAAM + ADSCs + HVFs group (defect repaired with HAAM scaffold pre-seeded with a 1:1 co-culture of ADSCs and HVFs). The cell-scaffold constructs for groups D-F were prepared as described in Section 2.6.

The modeling procedure was as follows: rats were anesthetized with isoflurane, and after abdominal skin disinfection, the skin was cut along the midline to expose the abdominal wall. A 2 cm × 2 cm area of the abdominal wall was excised to create the defect. The corresponding pre-cultured cell-scaffold composite (or HAAM scaffold alone for group C) was evenly applied to cover the defect area, ensuring full contact with the surrounding tissue, and fixed with 5–0 absorbable sutures before the incision was closed layer by layer. The day of modeling was designated as Day 0. Gross observation and photography of the implantation site were performed on Day 0, Day 7, and Day 14 (before sampling) to document wound healing and integration.

At the predefined endpoints (Day 7 and Day 14, with n = 4 per time point per group), rats were euthanized by deep anesthesia with isoflurane followed by cervical dislocation. The implanted construct along with the surrounding native tissue was harvested *en bloc*, fixed in 4% paraformaldehyde, and processed for subsequent histological and immunohistochemical analyses as described below.

### 2.8 HE staining

After sampling, the tissue specimens were fixed in 4% paraformaldehyde for 24 h, followed by dehydration through a graded series of ethanol (70%, 80%, 90%, and 100%) for 1 h each. After dehydration, the tissues were cleared in xylene and embedded in paraffin. The embedded tissues were sectioned into 4 μm thick slices using a microtome and mounted on glass slides. The sections were deparaffinized in xylene and rehydrated through a graded series of ethanol (100%, 95%, 80%, and 70%), followed by rinsing with distilled water. The sections were stained with hematoxylin for 5 min, rinsed with running water for 1 min, and then stained with eosin for 1 min. After rinsing with water, the sections were dehydrated through a graded series of ethanol (70%, 80%, 95%, and 100%), cleared in xylene, and mounted with neutral balsam. The sections were observed and photographed under a microscope.

### 2.9 Masson staining

The tissue specimens were fixed in 4% paraformaldehyde for 24 h, followed by dehydration, clearing, and paraffin embedding, using the same method as for HE staining. Sections of 4 μm thickness were deparaffinized and rehydrated, then stained with Weigert’s iron hematoxylin for 5 min and rinsed with running water for 1 min. The sections were stained with Biebrich scarlet-acid fuchsin solution for 5 min, rinsed with water, treated with phosphomolybdic acid solution for 5 min, and finally stained with aniline blue solution for 5 min, followed by rinsing with water. The sections were dehydrated through a graded series of ethanol (70%, 80%, 95%, and 100%), cleared in xylene, and mounted with neutral balsam. The distribution of collagen fibers (blue) and muscle fibers (red) was observed and photographed under a microscope.

### 2.10 Immunohistochemical (IHC) staining

The fixation, dehydration, embedding, and sectioning of tissue specimens were performed as described in Sections 2.8, 2.9. After deparaffinization and rehydration, the sections were incubated with 3% hydrogen peroxide solution for 10 min to block endogenous peroxidase activity, followed by rinsing with distilled water. Antigen retrieval was performed by microwaving the sections in citrate buffer (pH 6.0) for 10 min. After cooling and rinsing with PBS, the sections were blocked with 5% bovine serum albumin (BSA) for 30 min at room temperature.

For immunostaining, sections were incubated with specific primary antibodies at 4 °C overnight. The antibodies used were: rabbit anti-CD206 (1:200 dilution, Abcam, ab64693) to identify M2-like macrophages, rabbit anti-CD3 (1:200 dilution, Abcam, ab16669) as a pan-T cell marker, and mouse anti-CD68 (1:200 dilution, Abcam, ab955) as a general macrophage marker. Negative control sections were incubated with PBS instead of the primary antibody.

The following day, after thorough washing with PBS, the sections were incubated with corresponding HRP-labeled secondary antibodies (goat anti-rabbit or goat anti-mouse, 1:500, ZSGB-BIO) at room temperature for 30 min. After another PBS wash, color development was performed using a DAB chromogen kit (ZSGB-BIO) for 3–5 min, and the reaction was stopped with distilled water. Finally, the sections were counterstained with hematoxylin, dehydrated, cleared in xylene, and mounted with neutral balsam. The positive expression (brown precipitate) for CD206, CD3, and CD68 was observed and photographed under a light microscope. For semi-quantitative analysis, the number of positive cells in three random high-power fields (HPF, 400x) per sample was counted by two independent observers blinded to the group allocation.

### 2.11 RNA-Seq

On Day 14, tissue samples from the MODEL group and the ADSCs + HVFs group were collected, and total RNA was extracted using TRIzol reagent. The concentration and purity of RNA were measured using a Nanodrop, ensuring an A260/A280 ratio between 1.8 and 2.0. RNA integrity was assessed using an Agilent 2100 Bioanalyzer (RIN value >7.0). Subsequently, cDNA libraries were constructed using the TruSeq RNA Library Prep Kit and subjected to high-throughput sequencing on the Illumina NovaSeq 6000 platform. After quality control of the sequencing data, the reads were aligned to the rat reference genome (Rnor_6.0) using the STAR software, and differential expression analysis was performed using DESeq2 software (p<0.05, |log2FoldChange|>1). Significantly differentially expressed mRNAs were visualized using a heatmap.

### 2.12 GO-KEGG analysis

Functional enrichment analysis of significantly differentially expressed mRNAs obtained from RNA-Seq was performed using Gene Ontology (GO) and Kyoto Encyclopedia of Genes and Genomes (KEGG). The clusterProfiler software package was used to annotate the differential genes with GO terms, including Biological Process, Molecular Function, and Cellular Component. Simultaneously, KEGG pathway enrichment analysis was conducted to identify significantly enriched metabolic and signaling pathways (p<0.05). The enrichment results were presented as bar plots or bubble plots, with significantly enriched GO terms and KEGG pathways annotated with their functions and the number of involved genes.

### 2.13 GSEA analysis

To further explore the functional characteristics of differentially expressed genes, Gene Set Enrichment Analysis (GSEA) software was used to analyze the RNA-Seq data from the MODEL group and the ADSCs + HVFs group. Gene sets from the MSigDB database, including those related to ECM organization, collagen synthesis, and inflammatory responses, were selected as references. The enrichment score (ES) for each gene set was calculated using GSEA, and its significance was assessed (FDR<0.25). Significantly enriched gene sets were visualized as enrichment plots, and core enrichment genes were annotated in a heatmap. The GSEA results further validated the functional characteristics of differentially expressed genes in specific biological processes.

### 2.14 Annexin V-PI apoptosis assay

Cell apoptosis was assessed using the Annexin V-FITC/PI Apoptosis Detection Kit. HVFs were seeded in 6-well plates at a density of 1 × 10^5^ cells per well and divided into four experimental groups: (1) HVFs alone, (2) ADSCs + HVFs (co-cultured at 1:1 ratio using Transwell chambers), (3) ADSCs + HVFs + SB431542 (with 10 µM TGF-β inhibitor SB431542 added to the co-culture system), and (4) HVFs + TGFB2 (HVFs transfected with TGFB2 overexpression plasmid). After 48 h of treatment, cells were collected, washed with PBS, and resuspended in binding buffer. Annexin V-FITC (5 µL) and PI (5 µL) were added to the cell suspension, followed by incubation in the dark for 15 min at room temperature. Apoptosis rates were analyzed using a flow cytometer (BD FACSCalibur), and data were processed with FlowJo software.

### 2.15 Scratch wound healing assay

Cell migration ability was evaluated using the scratch wound healing assay. HVFs were seeded in 12-well plates at a density of 5 × 10^4^ cells per well and allowed to form a confluent monolayer. The cell monolayer was scratched vertically using a sterile 200 µL pipette tip. After washing with PBS to remove detached cells, fresh serum-free medium was added. The experimental groups included: (1) HVFs alone, (2) ADSCs + HVFs (co-cultured at 1:1 ratio using Transwell chambers), (3) ADSCs + HVFs + SB431542 (with 10 µM SB431542), and (4) HVFs + TGFB2 (TGFB2-overexpressing HVFs). Images of the scratch area were captured at 0h and 24 h using an inverted microscope (Nikon Eclipse Ti). The migration rate was calculated as: (Initial scratch area - Final scratch area)/Initial scratch area × 100%. Data were analyzed with ImageJ software.

### 2.16 Statistical analysis

All quantitative data are presented as mean ± Sprague Dawley (SD). The specific statistical tests applied to each dataset are detailed in the respective figure legends. In general, statistical comparisons among multiple groups were performed using one-way analysis of variance (ANOVA) followed by Tukey’s *post hoc* test for normally distributed data. For comparisons between two groups, the unpaired Student’s t-test was applied. For data that did not meet the assumptions of parametric tests (assessed by Shapiro-Wilk test), the non-parametric Kruskal–Wallis test followed by Dunn’s *post hoc* test (for multiple groups) or the Mann-Whitney U test (for two groups) was employed. A P-value of less than 0.05 was considered statistically significant. All statistical analyses were conducted using SPSS software (version 17.0) and GraphPad Prism (version 8.0).

## 3 Results

### 3.1 Successful isolation and characterization of key components for pelvic ligament equivalent construction

**FIGURE 1:**
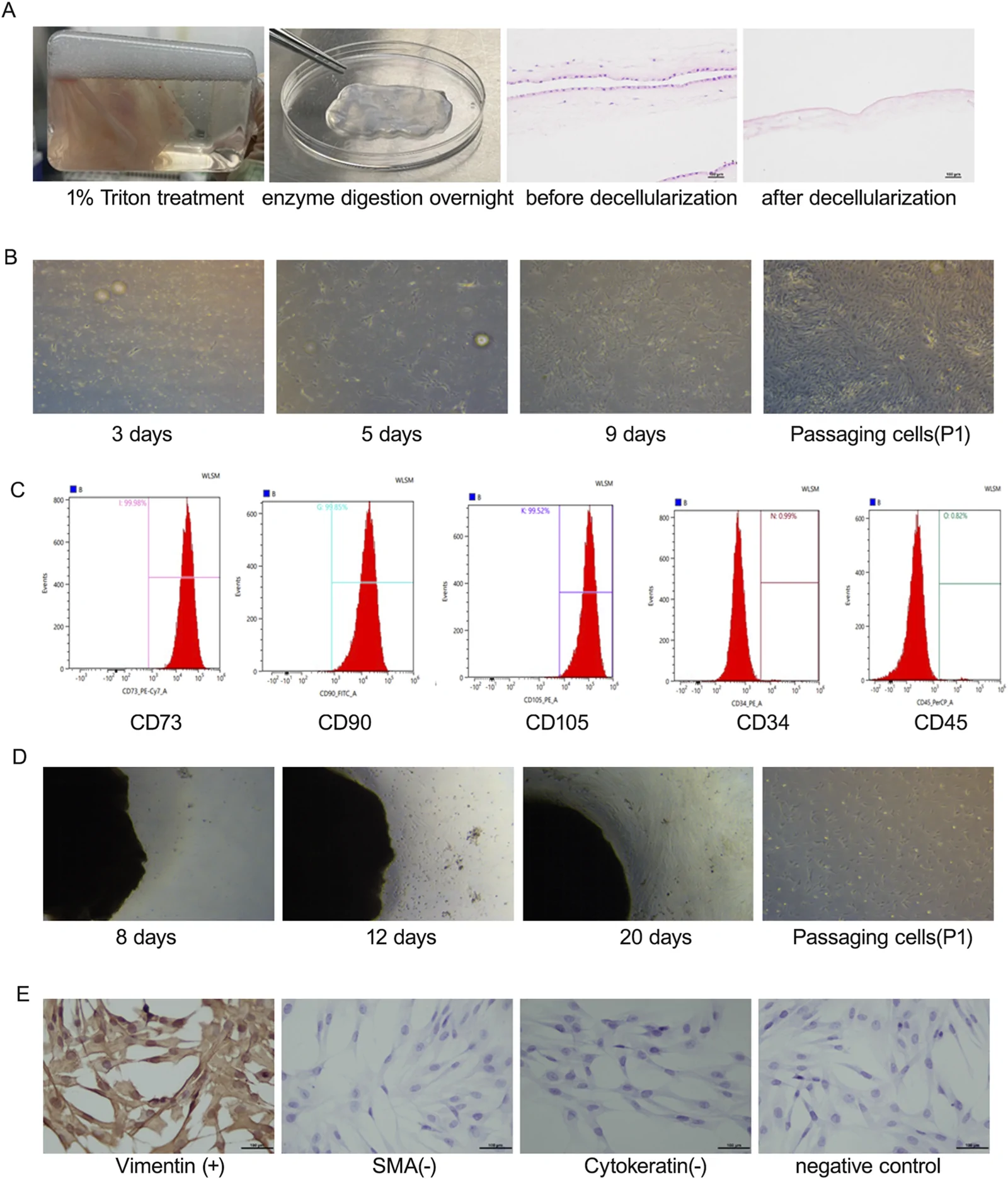
Successful Isolation and Characterization of Key Components for Pelvic Ligament Equivalent Construction. **(A)** HE staining of untreated and decellularized human amniotic membrane (HAAM) showing the removal of cell nuclei and preservation of ECM structure after treatment with 1% Triton X-100. **(B)** Light microscopy images of human adipose-derived mesenchymal stem cells (hADSCs) at different time points (days 3, 5, 9, and after first passage) demonstrating typical morphology and robust proliferation. **(C)** Flow cytometry analysis confirming hADSCs positive for CD73, CD90, and CD105, and negative for CD34 and CD45. **(D)** Phase-contrast images of human vaginal wall fibroblasts (HVFs) isolated from POP patients and non-POP controls at different time points (days 8, 12, 20, and after first passage), highlighting the patient-specific origin of the cells used in this study. **(E)** Immunocytochemical staining of HVFs showing positive expression of Vimentin and negative expression of α-SMA and Cytokeratin.

Decellularization of amniotic membrane with 1% Triton X-100 effectively removed cellular components while preserving ECM integrity. HE staining confirmed complete cell nuclei removal and intact ECM structure after treatment (Figure 1A). hADSCs isolated from POP patients exhibited a typical spindle-shaped, mesenchymal morphology and maintained this morphology through at least five serial passages (Figure 1B). Flow cytometry analysis verified the characteristic MSC phenotype, with over 95% of cells expressing CD73, CD90, and CD105, while less than 2% were positive for the hematopoietic markers CD34 and CD45 (Figure 1C). HVFs isolated from vaginal tissues demonstrated a typical elongated, fibroblast-like morphology (Figure 1D). Immunocytochemical staining quantitatively confirmed their fibroblast identity, with over 90% of cells positive for Vimentin and negative for α-SMA and Cytokeratin, while negative controls showed no staining (Figure 1E). These results establish the successful preparation and characterization of all essential components.

### 3.2 HAAM supports cell attachment, proliferation, and collagen restoration in co-culture systems

**FIGURE 2:**
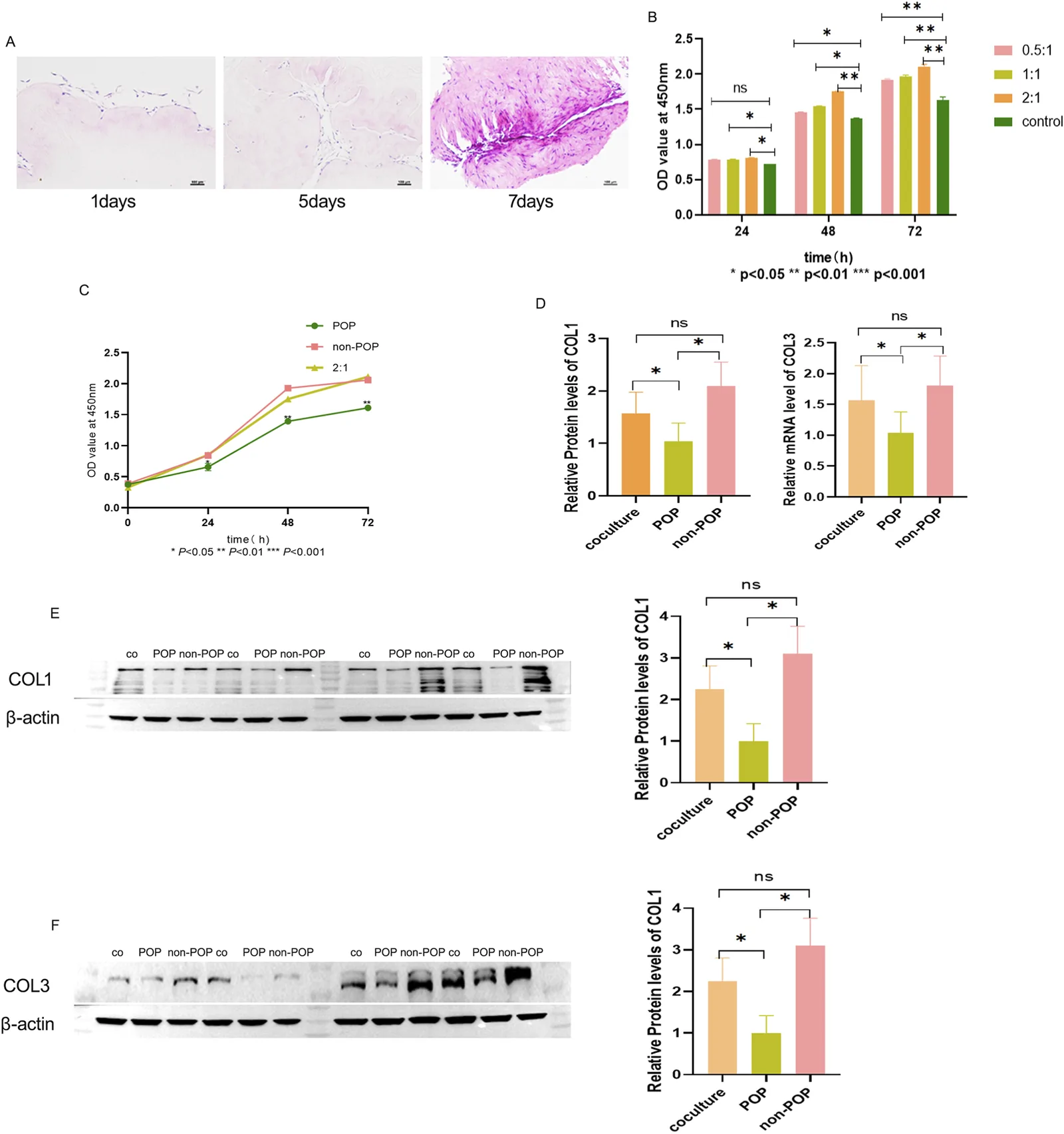
HAAM Supports Cell Attachment, Proliferation, and Collagen Restoration in Co-Culture Systems. **(A)** HE staining of HAAM scaffold with co-cultured ADSCs and HVFs (1:1 ratio) at days 1, 5, and 7, showing increased cell numbers and growth from the epithelial to stromal side. **(B)** CCK8 cell proliferation assay comparing HVFs proliferation under different co-culture gradients (ADSCs:HVFs = 0:1, 0.5:1, 1:1, 2:1) at 24h, 48h, and 72 h. Data are shown as mean ± SEM. *p < 0.05, **p < 0.01. **(C)** CCK8 growth curves comparing HVFs proliferation between POP and non-POP groups. **(D–F)** Western blot and qRT-PCR results showing protein and mRNA expression levels of COL1 and COL3 in POP, non-POP, and co-culture groups (2:1) at 72 h. Data are shown as mean ± SEM. *p < 0.05.

The 3D co-culture system with ADSCs and HVFs on HAAM scaffold demonstrated excellent biocompatibility. HE staining showed time-dependent cell growth from day 1–7, with cells migrating from the epithelial to the stromal side (Figure 2A). CCK-8 assays quantitatively revealed that ADSCs promoted HVFs proliferation in a dose- and time-dependent manner. Specifically, at 24h, the OD value of HVFs monoculture was 0.719 ± 0.001. Co-culture at ADSCs:HVFs ratios of 1:1 and 2:1 significantly increased OD values to 0.786 ± 0.005 and 0.812 ± 0.001, respectively (P < 0.05 vs. monoculture), while the 0.5:1 ratio (0.780 ± 0.008) showed no significant difference. This trend continued at 48 h and 72h, with the 2:1 ratio consistently yielding the highest OD values (e.g., 1.751 ± 0.007 at 48 h vs. 1.368 ± 0.010 for monoculture; 2.111 ± 0.026 at 72 h vs. 1.630 ± 0.043 for monoculture; all P < 0.05) (Figure 2B).

Notably, POP-derived HVFs exhibited significantly impaired basal proliferation compared to non-POP cells at all time points (e.g., 1.609 ± 0.006 vs. 2.057 ± 0.042 at 72h, P < 0.001). However, after 72 h of co-culture with ADSCs at a 2:1 ratio, the proliferation of POP-HVFs was restored to a level comparable to non-POP cells (OD value: 2.111 ± 0.029 vs. 2.057 ± 0.042) (Figure 2C).

Molecular analysis confirmed these functional improvements at the protein level. Western blot showed that the relative expression of COL1 in POP-HVFs (1.000 ± 0.414) was significantly lower than in non-POP cells (3.097 ± 0.698, P = 0.002). After co-culture, COL1 expression in POP-HVFs increased to 2.243 ± 0.570, showing no significant difference from the non-POP group (P = 0.099) but a significant increase from the untreated POP group (P = 0.012). A similar rescue effect was observed for COL3: expression in POP-HVFs (1.000 ± 0.353) was lower than in non-POP cells (1.590 ± 0.386, P = 0.038) and was significantly restored by co-culture to 1.843 ± 0.296 (P = 0.014 vs. POP group), reaching a level statistically indistinguishable from non-POP cells (P = 0.336) (Figures 2D–F). These data quantitatively demonstrate ADSCs' capacity to rescue both the proliferative defect and the deficient collagen production in POP-derived HVFs.

### 3.3 TGFB2 mediates ADSCs-induced functional improvements in HVFs

**FIGURE 3:**
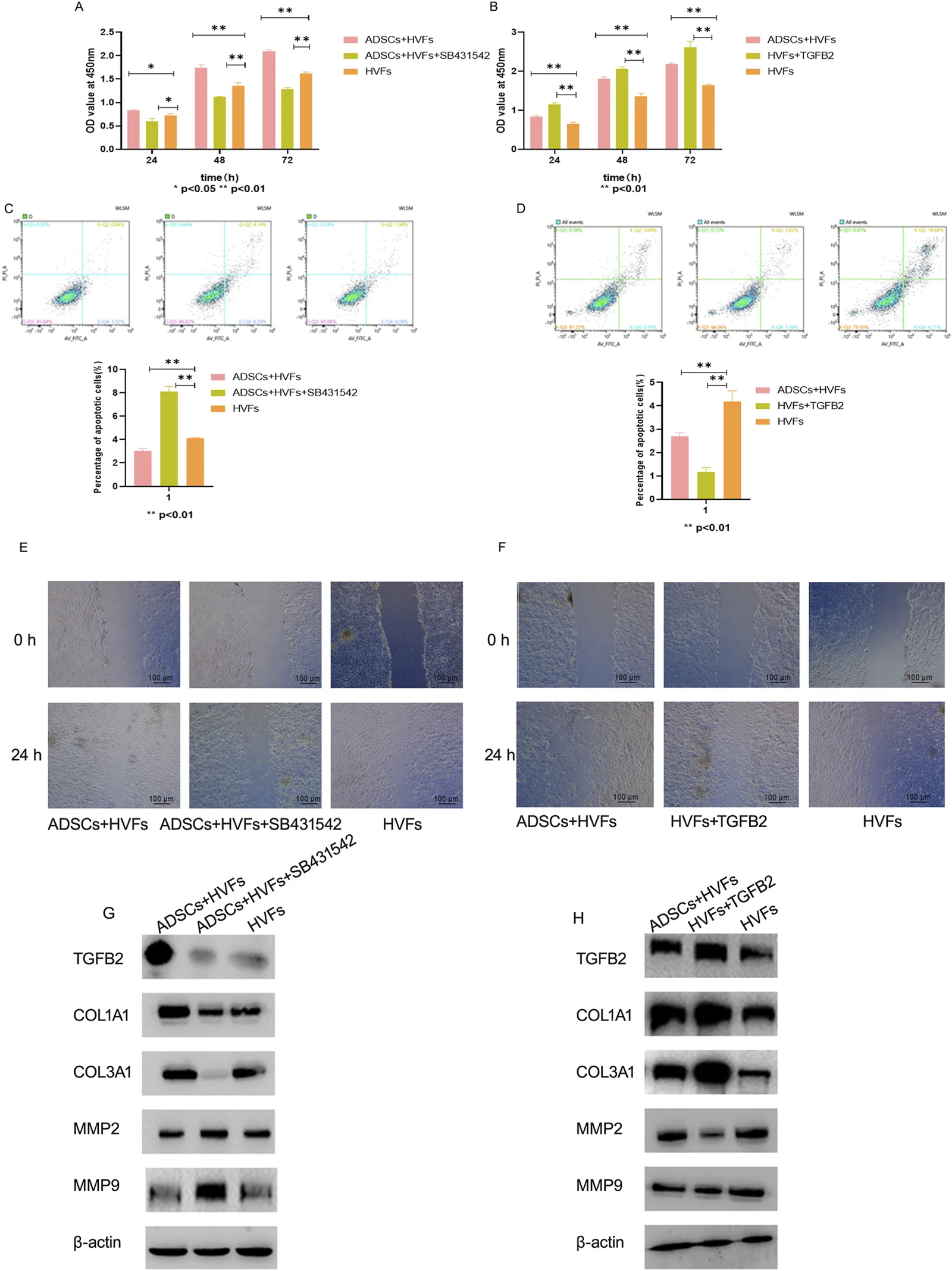
TGFB2 Mediates ADSCs-Induced Functional Improvements in HVFs. **(A)** CCK-8 assay showing proliferation of HVFs cultured alone or co-cultured with ADSCs in the presence or absence of TGF-β inhibitor SB431542 (10 μM). **(B)** CCK-8 assay showing proliferation of HVFs following TGFB2 overexpression. **(C)** Annexin V-PI apoptosis assay showing apoptotic rates of HVFs under different culture conditions. **(D)** Apoptosis assay showing the effect of TGFB2 overexpression on HVFs apoptosis. **(E)** Scratch wound healing assay showing migration ability of HVFs in different treatment groups. **(F)** Migration assay demonstrating the effect of TGFB2 overexpression on HVFs migration. **(G)** Western blot analysis showing protein expression levels of TGFB2, COL1A1, COL3A1, MMP2, and MMP9 in HVFs treated with or without SB431542. **(H)** Western blot analysis showing protein expression levels following TGFB2 overexpression.

To elucidate the molecular mechanism underlying the therapeutic effects of ADSCs, we investigated the role of TGFB2 using HVFs from POP patients. In loss-of-function experiments, ADSCs co-culture significantly enhanced HVFs proliferation (Figure 3A), inhibited apoptosis (Figure 3C), and promoted migration (Figure 3E) compared to HVFs alone. These beneficial effects were completely abolished by the TGFB2 inhibitor SB431542 (Figures 3A,C,E). Conversely, in gain-of-function experiments, direct TGFB2 overexpression in HVFs recapitulated the ADSCs co-culture phenotype, markedly promoting proliferation (Figure 3B), suppressing apoptosis (Figure 3D), and enhancing migration (Figure 3F).

Western blot analysis confirmed TGFB2 as the central mediator. Inhibition of TGFB2 signaling with SB431542 downregulated the expression of TGFB2 itself and its downstream ECM targets COL1A1 and COL3A1, while upregulating the matrix-degrading enzymes MMP2 and MMP9, thereby counteracting the ADSCs-induced changes (Figure 3G). Mirroring this, TGFB2 overexpression promoted the expression of TGFB2, COL1A1, and COL3A1, while suppressing MMP2 and MMP9 (Figure 3H), paralleling the effects of ADSCs co-culture. Collectively, these complementary data establish TGFB2 as the key paracrine factor through which ADSCs restore HVFs function.

### 3.4 Synergistic promotion of tissue repair and modulation of host immune response by HAAM-based constructs in a rat model

**FIGURE 4:**
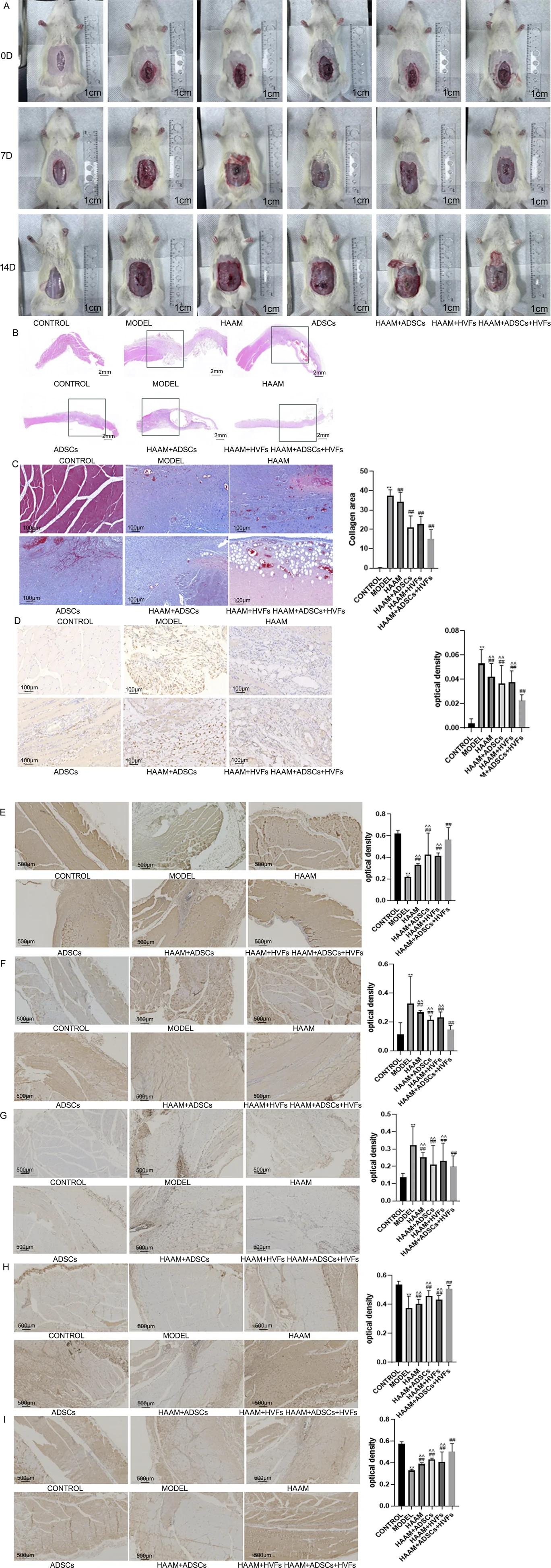
HAAM-based constructs synergistically promote tissue repair and modulate the host immune response in a rat abdominal wall defect model. **(A)** Gross observation of rat abdominal wall defect repair on Day 0, Day 7, and Day 14 in Blank Control, Model, HAAM, HAAM + ADSCs, HAAM + HVFs, and HAAM + ADSCs + HVFs groups (Scale bar: 1 cm). **(B)** Representative H&E staining of tissue sections on Day 14 showing tissue repair and integration in different groups (Scale bar: 2 mm). **(C,D)** Representative Masson’s trichrome staining showing collagen deposition (blue) and fibrosis levels **(C)**, and immunohistochemical (IHC) staining for CD206 (M2-like macrophage marker) expression levels **(D)** in different groups (Scale bars: 100 μm). **(E–I)** IHC staining for **(E)** α-SMA (myofibroblast marker) indicating the presence of activated, contractile cells within the neotissue, with the highest and most continuous expression observed in the HAAM + ADSCs + HVFs group **(F)** CD3 (pan-T cell marker) and **(G)** CD68 (general macrophage marker) to assess host inflammatory and immune cell infiltration **(H)** COL1A and **(I)** COL3A showing the expression and distribution of key structural collagens in the regenerated tissue. The HAAM + ADSCs + HVFs group exhibited the most intense and organized deposition for both collagens (Scale bars: 500 μm). *Data are shown as mean ± SEM. Statistical comparisons were performed using one-way ANOVA followed by Tukey’s *post hoc* test. *p < 0.01 vs. Control; ##p < 0.01 vs. Model; ^^p < 0.01 vs. HAAM + ADSCs + HVFs group.

We then validated the efficacy of the full composite *in vivo*. In a rat abdominal wall defect model, the HAAM + ADSCs + HVFs group demonstrated the most robust tissue regeneration, with complete wound coverage and seamless integration, outperforming all control groups in gross observation (Figure 4A). Histologically, this group showed superior tissue restoration with abundant, organized fibrous tissue and mature collagen deposition (Figures 4B,C), along with enhanced α-SMA^+^ myofibroblast activity (Figure 4E). The composite also optimally modulated the immune microenvironment, promoting a pro-regenerative state with reduced CD206^+^ M2-like macrophages (Figure 4D), without eliciting additional acute inflammation (comparable CD3^+^ T cell and CD68^+^ macrophage infiltration) (Figures 4F,G). Furthermore, immunohistochemistry confirmed the highest and most organized expression of COL1A and COL3A in the neotissue of the HAAM + ADSCs + HVFs group (Figures 4H,I).

### 3.5 Transcriptomic mechanisms of ECM remodeling and inflammation modulation

**FIGURE 5:**
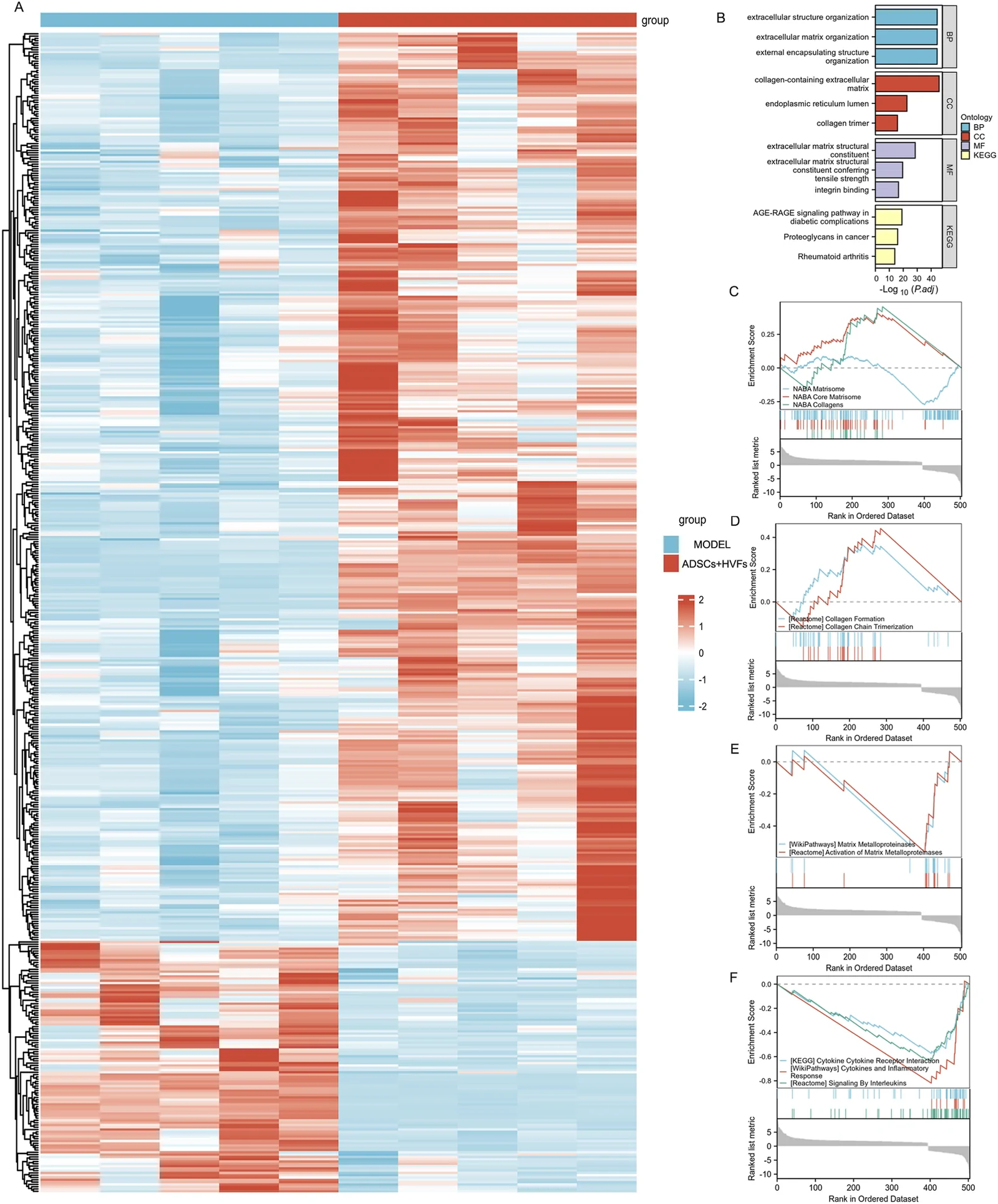
Mechanism of ADSCs and HVFs in Promoting Tissue Repair and Modulating ECM and Inflammation. **(A)** RNA-Seq analysis showing differentially expressed mRNAs between the ADSCs + HVFs group and the MODEL group. **(B)** GO-KEGG analysis highlighting enriched biological processes and pathways related to ECM organization, collagen synthesis, and inflammation. **(C)** GSEA analysis showing negative enrichment of NABA_MATRISOME and positive enrichment of NABA_CORE_MATRISOME and NABA_COLLAGENS. **(D)** GSEA analysis showing activation of collagen synthesis and processing-related pathways. **(E)** GSEA analysis showing inhibition of matrix metalloproteinase (MMP) function. **(F)** GSEA analysis showing suppression of inflammation and cytokine signaling pathways.

Compared to the Model group, the ADSCs + HVFs group showed 508 differentially expressed genes (DEGs), with strong upregulation of regenerative genes (e.g., TGFB2, COL1A1, COL3A1) and downregulation of catabolic and inflammatory genes (e.g., MMP2, MMP9, IL1B, IL6) (Figure 5A). Pathway analysis confirmed significant enrichment in ECM organization and wound healing processes, alongside suppression of key inflammatory pathways such as IL-17 signaling (Figure 5B). GSEA further supported coordinated ECM anabolism (enrichment of collagen synthesis pathways) (Figures 5C,D) and suppression of ECM degradation (inhibition of MMP-related pathways) (Figure 5E) and inflammation (downregulation of cytokine signaling) (Figure 5F). The complete list of DEGs is provided in Supplementary Table S1.

**FIGURE 6:**
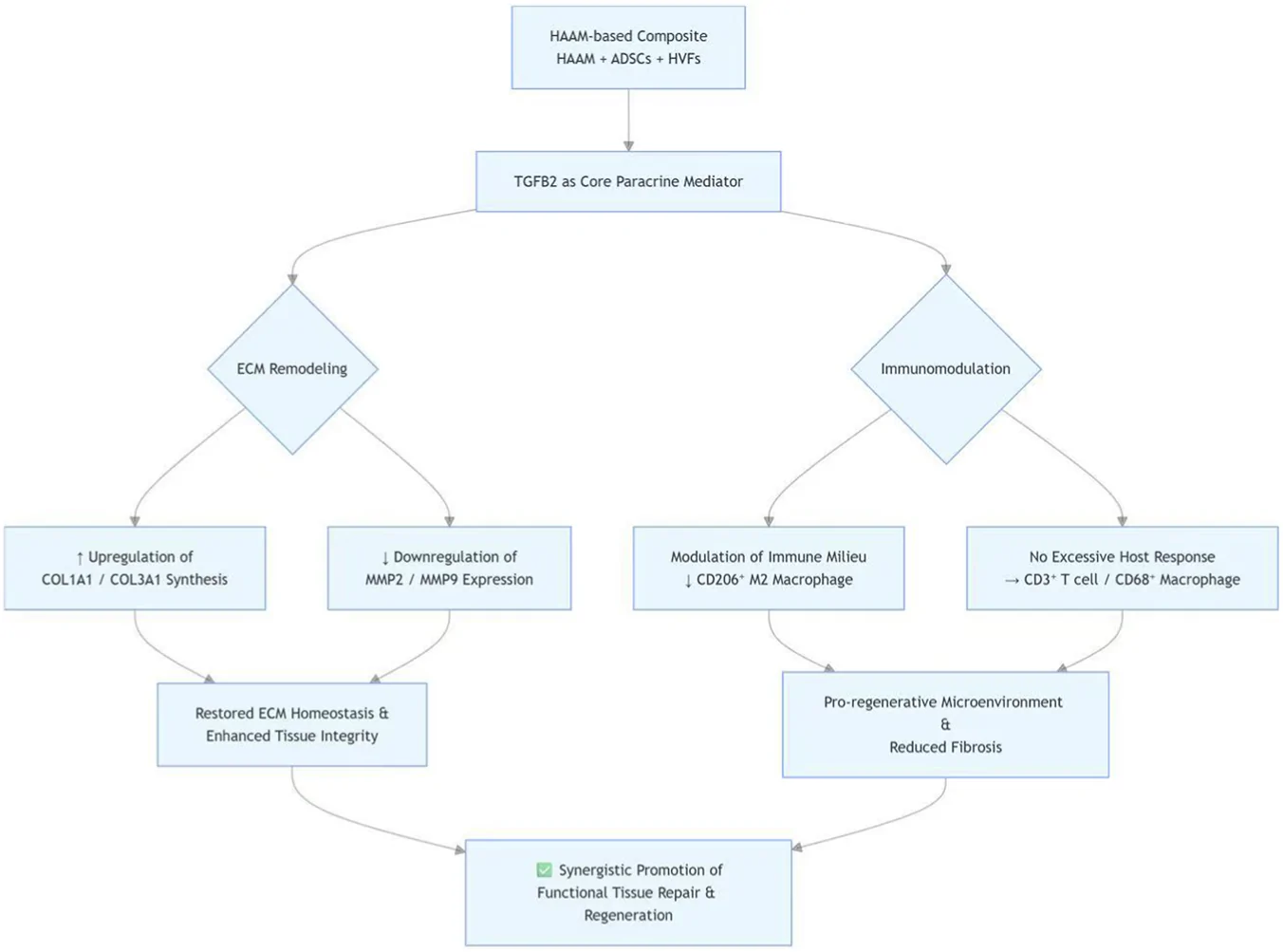
Proposed Mechanism of HAAM-Based Bioactive Composite in Pelvic Organ Regeneration. This schematic demonstrates the core mechanism by which adipose-derived stem cells (ADSCs) synergize with the HAAM scaffold to enhance pelvic tissue regeneration. ADSCs within the composite are activated to secrete high levels of transforming growth factor-beta 2 (TGFB2), which acts *via* paracrine signaling on co-cultured human vaginal fibroblasts (HVFs). This signaling axis drives multiple key regenerative processes: promoting HVF proliferation and migration, inhibiting cellular apoptosis, restoring extracellular matrix (ECM) homeostasis by upregulating collagen synthesis (COL1, COL3) and suppressing matrix metalloproteinase (MMP) activity (MMP2, MMP9), and sculpting a pro-regenerative local immune microenvironment. Together, these coordinated actions effectively restore tissue integrity and functional status, establishing a robust mechanistic basis for the clinical efficacy of the HAAM-based bioactive composite in pelvic organ prolapse treatment.

The integrated mechanism by which the HAAM-based composite promotes pelvic ligament regeneration is summarized in Figure 6.

## 4 Discussion

This study represents a significant advancement in the development of tissue-engineered pelvic ligament equivalents for treating POP. By combining ADSCs, prolapsed vaginal wall fibroblasts (HVFs), and decellularized human amniotic membrane (HAAM) into an integrated “HAAM-based bioactive composite,” we have established a novel therapeutic strategy that directly addresses the limitations of current POP treatments. Our findings collectively demonstrate that this approach promotes functional tissue repair, modulates the immune microenvironment, and restores structural integrity through multifaceted mechanisms. This biocompatible solution not only circumvents the complications associated with synthetic meshes but also harnesses the innate regenerative potential of autologous cells and natural biomaterials.

The successful establishment of key biological components forms the foundation of our approach. The decellularized HAAM scaffold preserved critical ECM architecture while eliminating cellular antigens, creating an ideal three-dimensional microenvironment for cell seeding and tissue regeneration (Ashouri et al., 2017; Wang et al., 2023; Dominici et al. 2006). The isolated ADSCs exhibited typical mesenchymal stem cell characteristics, while HVFs demonstrated authentic fibroblast phenotypes, ensuring their functional competence for tissue engineering applications. These findings align with the understanding that successful tissue regeneration requires synergistic interaction between functional seed cells and biocompatible scaffolds (Kim et al., 2007).

Our investigation revealed that the 3D co-culture system on HAAM scaffold significantly enhanced cellular behaviors and ECM remodeling. The dose-dependent promotion of HVFs proliferation by ADSCs and the successful restoration of COL1 and COL3 expression in POP-derived cells underscore the therapeutic potential of this combination at the cellular level. These observations corroborate previous studies demonstrating that ADSCs promote fibroblast proliferation and collagen synthesis through paracrine mechanisms (Yu et al., 2023). For example, ADSCs have been shown to secrete growth factors such as TGF-β and bFGF, which stimulate fibroblast activity and ECM production (Wu et al., 2023). The ability of HAAM to retain bioactive factors such as bFGF likely further enhanced this regenerative milieu (Budatha et al., 2011). This is particularly important in the context of POP, where ECM degradation and collagen imbalance are key pathological features (Du et al., 2025). By restoring collagen synthesis and ECM integrity, our approach addresses the underlying causes of POP and provides a durable solution for tissue repair.

The rat abdominal wall defect model provided compelling *in vivo* evidence for the superior efficacy of the combined therapy. We deliberately employed an immunocompetent Sprague Dawley (SD) rat model in a xenogeneic setting to stringently test two core hypotheses central to our study’s aims: first, that the ADSCs within our composite possess potent immunomodulatory capacity capable of mitigating potential host-versus-graft responses; and second, that the combined action of ADSCs and HVFs on the HAAM scaffold would drive functional tissue repair beyond what the scaffold alone or single-cell therapies could achieve. The selection of a 14-day endpoint for this proof-of-concept study was designed to critically evaluate the early-phase events crucial for implant success: initial host-scaffold integration, acute inflammatory response, and the onset of tissue remodeling. Critically, the inclusion of a “HAAM alone” control group allowed us to delineate the specific contributions of the cellular components. While the HAAM scaffold itself provided a conducive template for host cell infiltration and moderate repair, the HAAM + ADSCs + HVFs composite demonstrated significantly enhanced tissue integration, more mature and dense collagen deposition (as shown by Masson staining), and superior wound closure. This confirms a true synergistic effect between the bioactive scaffold and the dual-cell population.

Importantly, our data collectively provide strong evidence regarding the roles of ADSCs and HVFs in tissue healing and immune modulation, even without direct *in vivo* cell tracking. ADSCs are well-established modulators of inflammation: they secrete anti-inflammatory cytokines and promote macrophage polarization toward a pro-regenerative M2 phenotype (Kim and Hematti, 2009; Mollazadeh et al., 2017; Rahajeng, 2018). In our study, the HAAM + ADSCs + HVFs group exhibited the most pronounced reduction in CD206^+^ M2-like macrophages, indicating a more favorable immune microenvironment. This immunomodulatory effect, combined with the paracrine action of ADSCs (particularly through TGFB2) on HVFs, drives the enhanced ECM remodeling and tissue repair observed. HVFs, as the target cells in pelvic ligaments, are not merely passive recipients; their active participation in collagen synthesis and ECM organization is essential for achieving functional restoration. Our *in vitro* experiments directly showed that ADSCs rescue the impaired proliferation, migration, and ECM production of POP-derived HVFs, an effect that is mediated by TGFB2. While we did not perform *in vivo* tracking of transplanted human cells using an anti-human nuclear antibody, our histological and molecular assessments—including the lack of elevated T cell and macrophage infiltration compared to the HAAM alone group, combined with the robust ECM regeneration—strongly suggest that the human cells survived and actively contributed to tissue repair during the 14-day observation period. Nevertheless, we acknowledge that detailed cell fate mapping, such as using species-specific antibodies, would provide direct evidence of long-term cell persistence and distribution, and this remains an important avenue for future investigation.

RNA-Seq and pathway analysis confirmed that the HAAM + ADSCs + HVFs treatment modulates key biological processes involved in ECM organization, collagen synthesis, and inflammation. The upregulation of COL1A1, COL3A1, and TGFB2, along with the downregulation of MMP2 and MMP9, underscores the role of this treatment in promoting ECM stability and inhibiting degradation (Darzi et al., 2023). The suppression of inflammatory pathways such as IL-17 signaling further highlights its immunomodulatory effects (Alibabaei-Omran et al., 2024). Most importantly, our functional studies definitively established TGFB2 as the central mediator orchestrating ADSCs-induced therapeutic benefits on HVFs. Through complementary loss-of-function and gain-of-function approaches, we demonstrated TGFB2’s essential role in enhancing HVFs proliferation, suppressing apoptosis, promoting migration, and critically, in regulating ECM homeostasis by coordinately upregulating collagen synthesis and suppressing MMP expression.

Our findings extend previous research on stem cell therapy and decellularized scaffolds (Maher et al., 2013) by introducing a novel combinatorial approach specifically designed for POP treatment. In terms of clinical translation, the developed HAAM + ADSCs + HVFs composite is envisioned as a bioactive graft for procedures that require reinforcement or replacement of pelvic supportive ligaments. We propose a clinically feasible strategy: ADSCs would be obtained *via* minimally invasive lipoaspiration from the patient’s adipose tissue, and HVFs would be harvested from a small vaginal wall biopsy during the diagnostic or preparatory phase. The HAAM scaffold, sourced from donated placentas and processed into a decellularized, ‘off-the-shelf’ biomaterial, would serve as the universal carrier. This approach combines the safety and immunological compatibility of autologous cell transplantation with the practicality and immediate availability of an allogeneic natural scaffold. It holds particular promise for applications such as sacrocolpopexy or sacrospinous ligament fixation, where a robust, integrative, and remodellable suspensory material is needed to restore apical support. Compared to synthetic meshes (with risks of erosion and chronic pain) or high-recurrence native tissue repair, our HAAM-based composite approach offers distinct advantages: enhanced biocompatibility through autologous cells and a decellularized natural matrix; targeted addressing of POP pathological mechanisms *via* ECM restoration and immunomodulation; and the provision of temporary mechanical support by HAAM while facilitating its gradual replacement by functional neotissue.

Despite these promising results, several limitations warrant consideration and outline clear paths for future research. First and foremost, while our histological and molecular data strongly suggest functional improvement, the study lacks direct biomechanical assessment of the repaired tissue. Evaluating parameters such as tensile strength and elasticity of the regenerated abdominal wall in future studies is crucial to fully validate the functional restoration achieved by our composite. Second, the long-term stability, remodeling, and functional outcomes require investigation in larger animal models that more closely mimic the sustained mechanical loads and complex anatomy of the human pelvic floor. Third, while the SD rat model served as a valuable proof-of-concept for biocompatibility and early repair, future studies employing immunodeficient animals or more anatomically relevant pelvic floor defect models will be essential to precisely track the fate and function of the human cells and to model clinical translation more accurately. In particular, direct *in vivo* tracking using anti-human nuclear antibodies could provide definitive evidence for cell survival, migration, and contribution to neotissue formation, which would complement our current findings. Finally, potential heterogeneity in cell populations from different patients and the optimization of cell-scaffold ratios merit further exploration to standardize protocols for clinical application.

In conclusion, this study establishes a robust foundation for developing engineered pelvic ligament equivalents using a HAAM scaffold synergistically combined with ADSCs and HVFs. Our results demonstrate the composite’s ability to promote structural repair, modulate immune responses, and restore ECM integrity through TGFB2-mediated mechanisms. This comprehensive strategy addresses the multifactorial nature of POP pathogenesis and offers a promising regenerative alternative to current treatment modalities. The translation of this technology to clinical practice could potentially revolutionize POP management by providing a biologically integrated solution that aims to restore both anatomical support and long-term tissue homeostasis.

## Data Availability

The data presented in the study are deposited in the GEO repository, accession number GSE338232.

## Supplementary material

Supplementary Table S1 Complete list of differentially expressed genes (DEGs) in the ADSCs + HVFs group compared to the Model group.

## References

[B11] Ashouri S. Hosseini S. A. Hoseini S. J. Tara F. Ebrahimzadeh-Bideskan A. Webster T. J. (2017). Decellularization of human amniotic membrane for tissue engineering applications. J. Vis. Exp. (130), 56422. 10.1016/j.tice.2022.101818 35580526

[B30] Alibabaei-Omran F. Zabihi E. Seifalian A. M. Javanmehr N. Samadikuchaksaraei A. Gholipourmalekabadi M. (2024). Bilateral crosslinking with glutaraldehyde and 1-Ethyl-3-(3-Dimethylaminopropyl) carbodiimide: an optimization strategy for the application of decellularized human amniotic membrane in tissue engineering. J. Tissue Eng. Regen. Med. 2024, 8525930. 10.1155/2024/8525930 40225749 PMC11919163

[B1] Boreham M. K. Wai C. Y. Miller R. T. Schaffer J. I. Word R. A. (2002). Morphometric analysis of smooth muscle in the anterior vaginal wall of women with pelvic organ prolapse. Am. J. Obstet. Gynecol. 187 (1), 56–63. 10.1067/mob.2002.124843 12114889

[B2] Budatha M. Silva S. Montoya T. I. Suzuki A. Shah-Simpson S. Wieslander C. K. (2011). Dysregulation of protease and protease inhibitor expression in pelvic organ prolapse. J. Clin. Invest. 121 (7), 2454–2462. 10.1371/journal.pone.0056376

[B17] Cao J. Q. Liang Y. Y. Li Y. Q. Zhang H. L. Zhu Y. L. (2016). Adipose-derived stem cells enhance myogenic differentiation in the mdx mouse model of muscular dystrophy via paracrine signaling. Neural. Regen. Res. 11 (10), 1638–1643. 10.4103/1673-5374.193244 27904496 PMC5116844

[B3] Caplan A. I. Correa D. (2011). The MSC: an injury drugstore. Cell Stem Cell 9 (1), 11–15. 10.1016/j.stem.2011.06.008 21726829 PMC3144500

[B5] Chen B. H. Wen Y. li H. Polan M. L. (2016). Collagen metabolism and turnover in women with pelvic organ prolapse: a systematic review. Int. Urogynecol J. 13 (2), 80–87. 10.1007/s001920200020 12054187

[B6] Darzi S. Alappadan J. Paul K. Mazdumder P. Rosamilia A. Truong Y. B. (2023). Immunobiology of foreign body response to composite PLACL/gelatin electrospun nanofiber meshes with mesenchymal stem/stromal cells in a mouse model: Implications in pelvic floor tissue engineering and regeneration. Biomater. Adv. 155 213669. 10.1016/j.bioadv.2023.213669 37980818

[B8] Dominici M. Le Blanc K. Mueller I. Slaper-Cortenbach I. Marini F. Krause D. (2006). Minimal criteria for defining multipotent mesenchymal stromal cells. Cytotherapy 8 (4), 315–317. 10.1080/14653240600855905 16923606

[B32] Du J. Wu J. Song Q. Li S. Hong Y. Anwar A. (2025). Mesenchymal stem cell exosomes regulate TGFbeta/Smad3 by decreasing the METTL3-NEAT1 axis to inhibit scar progression after breast surgery. J Mol Histol. 56 (3), 161. 10.1007/s10735-025-10441-3 40392368

[B9] FDA 510(k) Premarket Notification K020643 (2002). Surgical Mesh for Transvaginal Repair of. U.S. Food and Drug Administration.

[B10] FDA. Urogynecologic Surgical Mesh (2019). Update on the Safety and Effectiveness of Transvaginal Placement for Pelvic Organ Prolapse. FDA Safety Communication.

[B12] Jackson S. R. Avery N. C. Tarlton J. F. Eckford S. D. Abrams P. Bailey A. J. (1996). Changes in metabolism of collagen in genitourinary prolapse. Lancet 347 (9016), 1658–1661. 10.1016/s0140-6736(96)91489-0 8642960

[B13] Kim J. Hematti P. (2009). Mesenchymal stem cell-educated macrophages: a novel type of alternatively activated macrophages. Exp. Hematol. 37 (12), 1445–1453. 10.1016/j.exphem.2009.09.004 19772890 PMC2783735

[B14] Kim W. S. Park B. S. Park S. H. Kim H. K. Sung J. H. (2009). Antiwrinkle effect of adipose-derived stem cell: activation of dermal fibroblast by secretory factors. J. Dermatol Sci. 53 (2), 96–102. 10.1016/j.jdermsci.2008.08.007 18829265

[B16] Kim W. S. Park B. S. Sung J. H. Yang J. M. Park S. B. Kwak S. J. (2007). Wound healing effect of adipose-derived stem cells: a critical role of secretory factors on human dermal fibroblasts. J. Dermatol. Sci. 48 (1), 15–24. 10.1016/j.jdermsci.2007.05.018 17643966

[B19] Liapis A. Bakas P. Pafiti A. Frangos-Plemenos M. Arnoyannaki N. Creatsas G. (2001). Changes of collagen type III in female patients with genuine stress incontinence and pelvic floor prolapse. Eur. J. Obstet. Gynecol. Reprod. Biol. 97 (1), 76–79. 10.1016/s0301-2115(00)00478-4 11435014

[B20] Maher C. Feiner B. Baessler K. Schmid C. (2013). Surgical management of pelvic organ prolapse in women. Cochrane Database Syst. Rev. (4), CD004014. 23633316 10.1002/14651858.CD004014.pub5

[B21] Mamede A. C. Carvalho M. J. Abrantes A. M. Laranjo M. Maia C. J. Botelho M. F. (2012). Amniotic membrane: from structure and functions to clinical applications. Cell Tissue Res. 349 (2), 447–458. 10.1007/s00441-012-1424-6 22592624

[B22] Mantovani A. Biswas S. K. Galdiero M. R. Sica A. Locati M. (2013). Macrophage plasticity and polarization in tissue repair and remodelling. J. Pathol. 229 (2), 176–185. 10.1002/path.4133 23096265

[B24] Moalli P. A. Shand S. H. Zyczynski H. M. Gordy S. C. Meyn L. A. (2005). Remodeling of vaginal connective tissue in patients with prolapse. Obstet. Gynecol. 106 (5 Pt 1), 953–963. 10.1097/01.AOG.0000182584.15087.dd 16260512

[B35] Mollazadeh S. Neshati V. Fazly Bazzaz B. S. Iranshahi M. Mojarrad M. Naderi-Meshkin H. (2017). Standardized sophora pachycarpa root extract enhances osteogenic differentiation in adipose-derived human mesenchymal stem cells. Phytother. Res. 31 (5), 792–800. 10.1002/ptr.5803 28337797

[B25] Niknejad H. Peirovi H. Jorjani M. Ahmadiani A. Ghanavi J. Seifalian A. M. (2008). Properties of the amniotic membrane for potential use in tissue engineering. Eur. Cell Mater. 15, 88–99. 10.22203/ecm.v015a07 18446690

[B33] Pang H. Zhang L. Han S. Li Z. Gong J. Liu Q. (2021). A nationwide population-based survey on the prevalence and risk factors of symptomatic pelvic organ prolapse in adult women in China - a pelvic organ prolapse quantification system-based study. BJOG. 128 (8), 1313–1323. 10.1111/1471-0528.16675 33619817 PMC8252658

[B15] Poncet S. Meyer S. Richard C. Aubert J.-D. Juillerat-Jeanneret L. (2005). The expression and function of the endothelin system in contractile properties of vaginal myofibroblasts of women with uterovaginal prolapse. Am. J. Obstet. Gynecol. 192 (2), 426–432. 10.1016/j.ajog.2004.09.018 15695982

[B18] Rahajeng R. (2018). The increased of MMP-9 and MMP-2 with the decreased of TIMP-1 on the uterosacral ligament after childbirth. Pan Afr. Med. J. 30, 283. 10.11604/pamj.2018.30.283.9905 30637068 PMC6317396

[B4] Raya-Rivera A. M. Esquiliano D. Fierro-Pastrana R. López-Bayghen E. Valencia P. Ordorica-Flores R. (2014). Tissue-engineered autologous vaginal organs in patients: a pilot cohort study. 10.1016/S0140-6736(14)60542-0 24726478

[B27] Ren G. Zhang L. Zhao X. Xu G. Zhang Y. Roberts A. I. (2008). Mesenchymal stem cell-mediated immunosuppression occurs *via* concerted action of chemokines and nitric oxide. Cell Stem Cell 2 (2), 141–150. 10.1016/j.stem.2007.11.014 18371435

[B28] Takacs P. Gualtieri M. Nassiri M. Candiotti K. Medina C. A. (2008). Vaginal smooth muscle cell apoptosis is increased in women with pelvic organ prolapse. Int. Urogynecol J. 19 (11), 1559–1564. 10.1007/s00192-008-0690-z 18677435

[B23] Wang L. Han L. Y. Li H. L. (2023). Etiological study of pelvic organ prolapse and stress urinary incontinence with collagen status and metabolism. Zhonghua Yi Xue Za Zhi. 93 (7), 500–3. 23660316

[B29] Wu M. Yu Z. Matar D. Y. Karvar M. Chen Z. Ng B. (2023). Human amniotic membrane promotes angiogenesis in an oxidative stress chronic diabetic murine wound model. Adv Wound Care (New Rochelle). 12 (6), 301–315. 10.1089/wound.2022.0005 35293255

[B31] Wu J. M. Hundley A. F. Fulton R. G. Myers E. R. (2009). Forecasting the prevalence of pelvic floor disorders in U.S. women: 2010 to 2050. Obstet. Gynecol. 114 (6), 1278–1283. 10.1097/AOG.0b013e3181c2ce96 19935030

[B7] Yu H. Wu Y. Zhang B. Xiong M. Yi Y. Zhang Q. (2023). Exosomes derived from E2F1^–/–^ adipose-derived stem cells promote skin wound healing via mir-130b-5p/TGFBR3 axis. Int. J. Nanomedicine. 18 6275–6292. 10.2147/IJN.S431725 37941530 PMC10629453

[B34] Zuk P. A. Zhu M. Mizuno H. Huang J. Futrell J. W. Katz A. J. (2001). Multilineage cells from human adipose tissue: implications for cell-based therapies. Tissue Eng. 7 (2), 211–228. 10.1089/107632701300062859 11304456

