# Correction: TGFB2-mediated regeneration of pelvic ligament equivalents using a stem cell-fibroblast-decellularized membrane composite

**DOI:** 10.3389/fbioe.2026.1983762

**Published:** 2026-09-17

**Authors:** Yingying Dong, Yu Zhang, Yinan Duan, Zhijun Xia

**Affiliations:** 1 Department of Obstetrics and Gynecology, Shengjing Hospital of China Medical University, Shenyang, Liaoning, China; 2 Department of Gynecology, Liaoning Maternal and Child Health Hospital, Shenyang, Liaoning, China; 3 Department of Research and Development, Shenyang Engineering Technology R&D Center of Cell Therapy Co., Ltd., Shenyang, Liaoning, China; 4 Department of Gynecology, Obstetrics and Gynecology Hospital of Fudan University, Shanghai, China

**Keywords:** adipose-derived stem cells, decellularized human amniotic membrane, extracellular matrix, pelvic organ prolapse, tissue engineering

An incorrect **Funding** statement was provided. The correct funder is National Natural Science Foundation.

An incorrect number was provided for the role and mechanism of miR-23a−3p/EGR−1 in regulating the senescence of vaginal wall fibroblasts. The correct number is 82271613.

An incorrect number was provided for Establishment of preclinical evaluation system and clinical trial of fully degradable pelvic floor repair patch. The correct number is 2023YFC2411205.

The original version of this article has been updated.

